# Progress in Neurology

**Published:** 1901-11-23

**Authors:** 


### PROGRESS IN NEUROLOGY.

(*Continued from page* 119.)

**Hyperpyrexia.**—Halliburton[1] in his third lecture on the Chemical Side of Nervous Activity discussed the chemical pathology of hyperpyrexia. The physico-chemical cause of death in this condition is heat coagulation of cell globulin. When this constituent of cell-protoplasm is coagulated the vitality of protoplasm is destroyed, just as muscle loses its irritability when the corresponding proteid in that tissue is coagulated. The temperature at which such coagulation is readily produced is 117° F., but it will occur at a temperature of 108° F. if long continued. The chemical change in the brain substance is coincident with the histological (chromatolytic) changes in the nerve cells which can be rendered evident by the methylene-blue process. The expression "coagulation-necrosis" employed by Marinesco for this appearance is therefore justifiable.

**In General Paralysis** the cerebro-spinal fluid is much increased in quantity, and takes the place of the atrophied brain material. It contains a large amount of nucleo-proteid and of choline which doubtless originate from the disintegration of the brain tissue; whereas normal cerebro-spinal fluid does not contain nucleo-proteid, and the amount of choline is so small that it cannot be readily identified. Choline when injected into an animal produces a fall in the arterial blood-pressure, mainly due to dilatation of the peripheral vessels, though partly by its action on the heart. In cases of general paralysis the choline produced by the disintegration of the brain passes into the blood, where it can be detected during convulsive seizures. Possibly then some of the symptoms of general paralysis may be due to auto-intoxication. Choline can be found in the blood in various other degenerative diseases—such as combined sclerosis, disseminated sclerosis, alcoholic neuritis, and beri-beri. A similar condition of the blood can be produced artificially in cats by a division of both sciatic nerves, and is most marked in those animals in which the degenerative process is at its height, as tested histologically by the Marchi reaction. The Marchi reaction is the black staining produced by Marchi's fluid—a mixture of Müller's fluid and osmic acid—after the tissue has been previously hardened in Müller's fluid. On the other hand Marchi's fluid stains healthy medullary sheaths greenish-grey, and it stains ordinary adipose tissue black. Now chemical analysis shows that whereas the medullary sheaths of healthy nerves contain a phosphorised fat (lecithin), ordinary adipose tissue contains a non-phosphorised fat. The chemical explanation then of the Marchi reaction is the replacement of phosphorised by a non-phosphorised fat. In Wallerian degeneration the lecithin which forms the main constituent of myelin is broken up into its four constituents, viz., (1) choline—which can be detected for a time in the blood ; (2) phosphoric acid—this disappears next ; and (3 and 4) fatty acid and glycerine—are the elements of neutral fat, which, like other neutral fats, gives the black Marchi reaction. In time, however, this is removed also.

[1] Lancet. June 22, 1901.

